# A heterobimetallic copper–titanium oxo cluster with a new structural motif

**DOI:** 10.1007/s00706-015-1558-9

**Published:** 2015-09-01

**Authors:** Christine Artner, Ayse Koyun, Ulrich Schubert

**Affiliations:** Institute of Materials Chemistry, Vienna University of Technology, Getreidemarkt 9, 1060 Vienna, Austria

**Keywords:** Metal oxo clusters, Copper compounds, Titanium compounds

## Abstract

**Abstract:**

Mixed-metal oxo clusters Cu_4_Ti_5_O_6_(OOCR)_16_ (OOCR = methacrylate, propionate) were obtained by reaction of titanium alkoxides and copper carboxylates with propionic or methacrylic acid.

**Graphical abstract:**

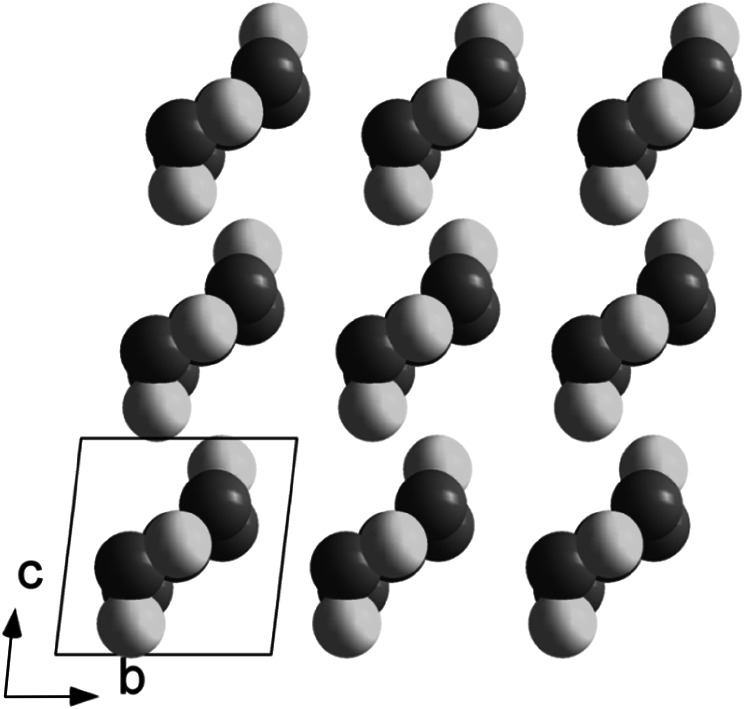

## Introduction

Metal oxo clusters can be obtained by controlled hydrolysis of metal alkoxides and regarded as intermediates or building blocks in the formation of sol–gel materials. They may also serve as model compounds for structural units in amorphous gels. Especially for bimetallic gels, nearly nothing is known about distribution and mutual arrangement of the metals. The structural characterization of heterobimetallic oxo clusters may therefore provide some evidence in this respect.

Schubert et al. have previously developed a convenient approach to synthesize heterobimetallic oxo clusters with early transition metals (Ti, Zr, Y) as one of the metals. To this end, carboxylic acids were initially reacted with metal alkoxide mixtures. The oxo groups of the cluster core are produced in situ through esterification of the carboxylic acid with alcohol which is liberated by substitution of alkoxo ligands against carboxylate ligands. A simplification of this method is to use only one metal alkoxide, and a metal acetate as the source for the second metal [1–3].

The structures of most titanium-containing heterobimetallic clusters are based on a few common motifs, despite variation of coordination number, ionic size, and charge of the second metal. Some clusters with Pb, Sr [1], La, and Ce [2] are structurally derived from cyclic Ti_8_O_8_(OOCR)_16_. Another series is based on partial replacements of the Ti atoms in Ti_6_O_4_(OR)_8_(OOCR’)_8_ by Fe, Zn, Cd, Ca, or Sr, with concomitant rearrangement of the ligand shell [3]. The structures of clusters with Zr [4], Hf [5], Y [6], Sm, Eu, Gd, or Ho [2] are zig-zag chains of condensed [TiO_6_] and [MO_x_] polyhedra. In this article, we describe a Cu/Ti oxo cluster the structure of which is not related to these series although the radius and coordination properties of Cu^2+^ ions are not too different to that of other divalent metals used before.

## Results and discussion

**Fig. 1:**
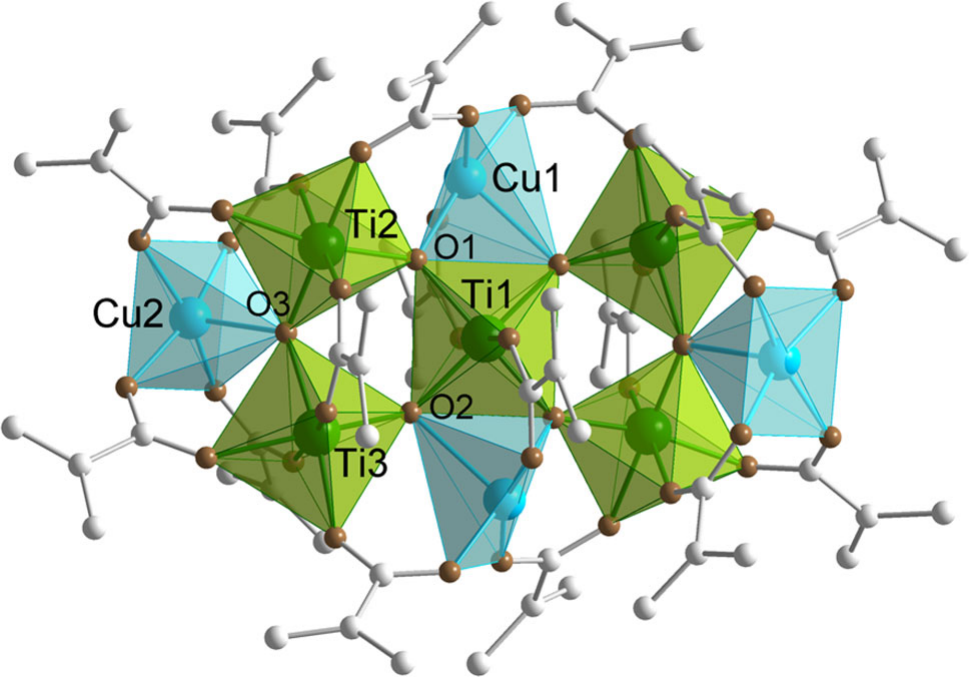
Molecular structure of Cu_4_Ti_5_O_6_(OMc)_16_ (**1**). Hydrogen atoms have been omitted for clarity. Cu1-O1 1.9592(12), Cu1-O2 2.3446(12), Cu1-O4 1.9687(13), Cu1-O6 1.9427(13), Cu1-O8 1.9289(13), Cu2-O3 2.1984(12), Cu2-O10 1.9742(14), Cu2-O12 1.9401(14), Cu2-O14 1.9598(14), Cu2-O16 1.9333(14), Ti1-O1 1.9179(11), Ti1-O2 1.9205(12), Ti1-O5 1.9958(12), Ti2-O1 1.8503(12), Ti2-O3 1.7993(12), Ti2-O7 2.0623(13), Ti2-O11 2.0260(13), Ti2-O13 1.9960(12), Ti2-O18 2.0081(13), Ti3-O2 1.7656(12), Ti3-O3 1.9066(12), Ti3-O9 2.0014(13), Ti3-O15 1.9388(13), Ti3-O17 2.1081(13), Ti3-O19 2.0131(13) Å

When Ti(O*i*Pr)_4_, Ti(OPr)_4_, or Ti(OBu)_4_ were reacted with Cu(OAc)_2_ and methacrylic acid (HOMc) in different molar ratios, the centrosymmetric heterobimetallic cluster Cu_4_Ti_5_(*μ*_3_-O)_6_(*μ*_2_-OMc)_16_ (**1**, Fig. 1) was obtained. The Cu:Ti and metal:acid ratio did not influence the formation of **1**. Some solid Cu(OAc)_2_ remained unreacted, however, even for a Cu:Ti ratio of 4:5 and a large excess of methacrylic acid. The formation of known Ti_8_O_8_(OMc)_16_ [1, 7] was supressed by a greater excess of methacrylic acid. The isostructural cluster Cu_4_Ti_5_O_6_(OProp)_16_ (**2**) was obtained from Ti(O*i*Pr)_4_, Cu(OAc)_2_, and propionic acid (HOProp).

The Ti atoms in **1** and **2** are octahedrally coordinated by six oxygen atoms, the coordination geometry of the copper atoms is a square pyramid. The [TiO_6_] and [CuO_5_] polyhedra are connected through six *μ*_3_-oxygen atoms, each of which being bonded to two Ti and one Cu atom. The five Ti atoms and the core oxygen atoms are coplanar; the copper atoms are located above and below the Ti_5_O_6_ plane. While the [TiO_6_] octahedra are corner-sharing among each other and form a face-centred rectangle, two [CuO_5_] square pyramids (Cu2) share a corner with two [TiO_6_] octahedra and the other two share an edge with that of central Ti1 (which is located on an inversion centre). The central Ti atom is thus coordinated to four *μ*_3_-O, the other Ti atoms to two *μ*_3_-O, and the Cu atoms to either one or two.

The apical Cu–O distances are longer [Cu1-O2 2.345(1), Cu2-O3 2.198(1) Å] than the equatorial [1.929(1)–1.974(1) Å], and therefore the *μ*_3_-oxygen atoms O2 and O3 are unsymmetrically surrounded by the metal atoms. This also influences the Ti-*μ*_3_-O distances.

The methacrylate ligands bridge either two Ti atoms, or a Ti and a Cu atom. Two OMc ligands bridge the central Ti atom with Cu1 on opposite sides of the Ti_5_O_6_ plane. Two OMc ligands bridge Cu2 with Ti2 and Ti3 each. The four oxygen atoms of the bridging ligands form the basal plane of the square pyramid of Cu2. OMc ligands bridge Cu1 to each of its neighbouring Ti atoms. The Cu–O distances of the ligands are in the range 1.929(1)–1.974(1) Å. The Ti–O bond lengths are in the range 1.939(1)–2.026(1) Å, with only two outliers.

**Fig. 2:**
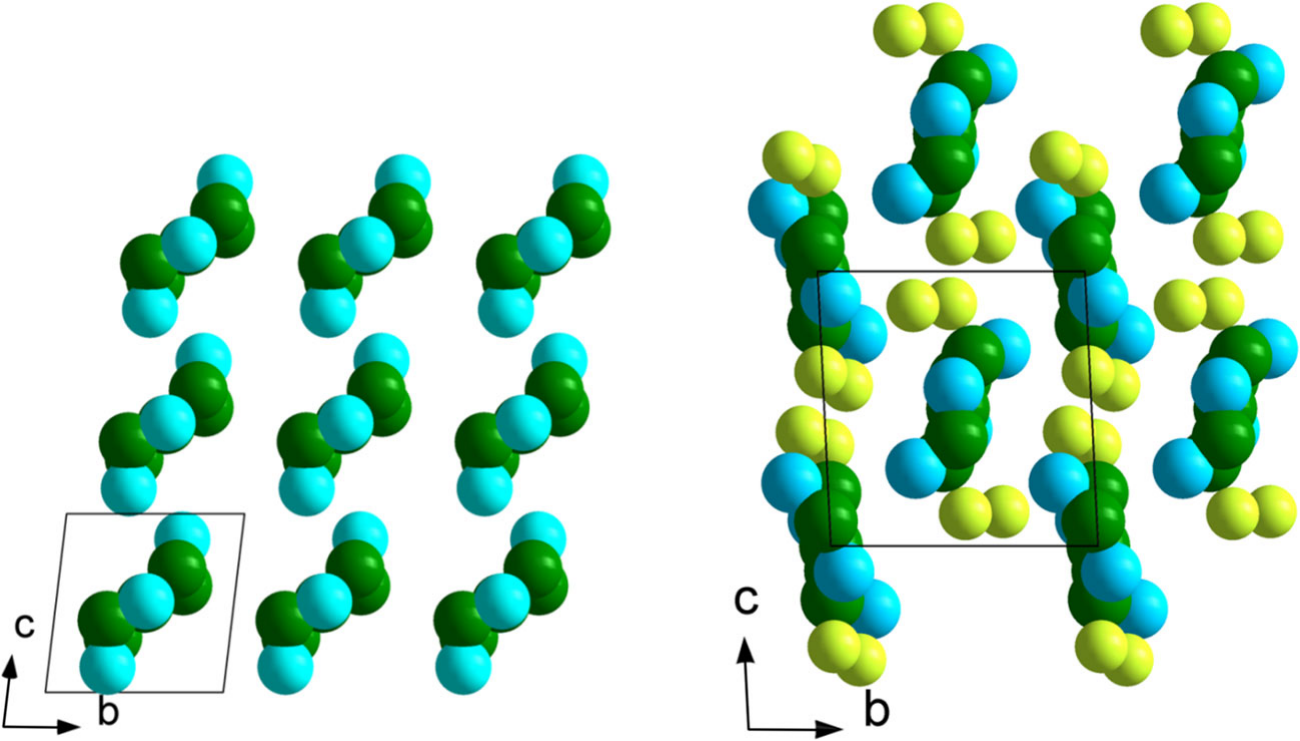
Crystal structure of Cu_4_Ti_5_O_6_(OMc)_16_ (**1**, *left*) and Cu_4_Ti_5_O_6_(OMc)_16_·CH_2_Cl_2_ (**1S**, *right*). Only metal and chlorine atoms were drawn in a space filling model

Cu_4_Ti_5_(*μ*_3_-O)_6_(*μ*_2_-OMc)_16_ was also obtained with co-crystallized CH_2_Cl_2_ (**1S**). The molecular structure is the same as that of **1**, but the crystal structure is different (Fig. 2).

## Conclusions

The structures of Cu_4_Ti_5_O_6_(OOCR)_16_ represent a new structural motive, which cannot be derived from known Ti oxo clusters in a straightforward manner. There is a faint resemblance to the structure of Zn_2_Ti_4_O_4_(O*i*Pr)_2_(OMc)_10_ [3], where two [ZnO_4_] tetrahedra are connected to a ring of four corner-sharing [TiO_6_] octahedra through two *μ*_3_-O groups. The structure of **1** and **2** is obtained by cutting the Zn/Ti cluster in two halves, replacing the [ZnO_4_] tetrahedra by [CuO_5_] square pyramids and inserting the rod-shaped unit Cu1–Ti1–Cu1* unit between both halves.

Formation of oxo clusters by reaction of metal alkoxides and carboxylic acids usually depends to a considerable degree on the OR groups of the employed metal alkoxide as well as the kind of carboxylic acid. This is surprisingly not the case for the Ti/Cu cluster type reported in this communication. This cluster type therefore appears to be a thermodynamically favoured structural entity. Another unusual feature is that the cluster contains no residual OR ligands, as in most other titanium/metal oxo clusters isolated until present. Both features may be connected with each other.

## Experimental

All experiments were carried out under Ar atmosphere using standard Schlenk techniques. Ti(OPr)_4_, Ti(OBu)_4_, and Cu(OAc)_2_ were obtained from Aldrich, Ti(O*i*Pr)_4_ from ABCR. Water-free copper acetate was obtained by drying in vacuum at 130 °C over night. The drying process was monitored by IR spectroscopy. Cu(OMc)_2_ was synthesized according to literature [8].

### Cu_4_Ti_5_ clusters

#### Cu_4_Ti_5_O_6_(OMc)_16_ (**1**, C_64_H_80_Cu_4_O_38_Ti_5_)

Dry Cu(OAc)_2_ (0.363 g, 2 mmol) and 1.137 g of Ti(O*i*Pr)_4_ (4 mmol) were reacted with 2.32 g of methacrylic acid (27 mmol). The Cu(OAc)_2_ did not dissolve completely, even after stirring for 24 h. The solution was then filtered; dark green crystals of **1** formed after 1 week from the filtrate. Yield 0.28 g (21 % rel. Cu). After another week white crystals Ti_8_O_8_(OMc)_16_ [1] were also formed in the same solution.

#### Cu_4_Ti_5_O_6_(OMc)_16_·CH_2_Cl_2_ (**1S**, C_66_H_84_Cl_4_Cu_4_O_38_Ti_5_)

Reaction of 0.935 g of Cu(OMc)_2_ (4 mmol) with 1.42 g of Ti(O*i*Pr)_4_ (5 mmol) and 3.10 g of methacrylic acid (36 mmol) resulted in the same cluster, but with a different crystal structure due to the incorporation of CH_2_Cl_2_ which originates from the synthesis of Cu(OMc)_2_.

#### Cu_4_Ti_5_O_6_(OProp)_16_ (**2**, C_48_H_80_Cu_4_O_38_Ti_5_)

Dry Cu(OAc)_2_ (0.182 g, 1 mmol) and 0.284 g of Ti(O*i*Pr)_4_ (1 mmol) were reacted with 1.44 g of propionic acid (24 mmol). The solution was clear and blue after 2 days of stirring. The solution was cooled to −18 °C and green crystals of **2** were isolated after 2 week. Yield 0.29 g (25 % rel. Cu).

### X-ray crystallography

Crystallographic data were collected on a Bruker AXS SMART APEX II four-circle diffractometer with κ-geometry at 100 K using MoK_α_ (*λ* = 0.71073 Å) radiation. The data were corrected for polarization and Lorentz effects, and an empirical absorption correction (SADABS) was employed. The cell dimensions were refined with all unique reflections. SAINT PLUS software (Bruker Analytical X-ray Instruments, 2007) was used to integrate the frames. Symmetry was checked with the program PLATON.

The structures were solved by charge flipping (JANA2006). Refinement was performed by the full-matrix least-squares method based on *F*^2^ (SHELXL97 [9]) with anisotropic thermal parameters for all non-hydrogen atoms. Hydrogen atoms were inserted in calculated positions and refined riding with the corresponding atom. Crystal data, data collection parameters, and refinement details are listed in Table 1. Some propionate ligands of **2** show positional disorder.

**Table 1:** Crystal data, data collection parameters, and refinement details

	**1**	**1S**	**2**
Empirical formula	C_64_H_80_Cu_4_O_38_Ti_5_	C_66_H_84_Cl_4_Cu_4_O_38_Ti_5_	C_48_H_80_Cu_4_O_38_Ti_5_
*M*_r_	1950.94	2120.79	1758.78
Crystal system	Triclinic	Triclinic	Monoclinic
Space group	*P* $\bar{1}$	*P* $\bar{1}$	*P*2_1_/*n*
*a*/Å	12.6918(3)	13.8581(9)	15.7865(9)
*b*/Å	12.7251(3)	17.568(1)	13.6433(9)
*c*/Å	13.5794(4)	18.426(1)	16.532(1)
*α*/°	91.584(1)	92.384(2)	90
*β*/°	69.965(1)	101.229(2)	108.080(4)
*γ*/°	84.063(1)	91.658(2)	90
*V*/Å^3^	2034.87(9)	4395.4(5)	3384.9(4)
*Z*	1	2	2
*D*_*x*_/g cm^−3^	1.592	1.602	1.726
*µ*/mm^−1^	1.575	1.583	1.882
Crystal size/mm	0.38 × 0.33 × 0.30	0.50 × 0.32 × 0.14	0.29 × 0.25 × 0.22
*T*_min_, *T*_max_	0.5860, 0.6495	0.5050, 0.8088	0.6113, 0.6822
No. measd, indep, obs. refl. (*I* > 2*σ*(*I*))	46,053, 9380, 8193	307,797, 36,951, 28,747	134,762, 12,959, 9150
*θ*_max_/°	27.56	34.42	33.25
*R*[*F* ^*2*^ > 2*σ*(*F*)]*, ωR*(*F* ^*2*^), *S*	0.0260, 0.0705, 1.050	0.0342, 0.0864, 1.088	0.0486, 0.0943, 1.114
No. of parameters	510	1100	466
Weighting scheme*	*x* = 0.0328, *y* = 1.7735	*x* = 0.0275, *y* = 4.6424	*x* = 0.0298, *y* = 3.7190
*δρ*_max_, *δρ*_min_/e Å^−3^	0.463, −0.340	1.051, −0.992	0.932, −1.034

* *ω* = 1/[*σ* ^*2*^(*F*_*0*_^*2*^) + (*xP*)^2^ + *yP*], where *P* = (*F*_*0*_^*2*^ +2*F*_*c*_^2^)/3

CCDC 1051410 (**1**), 1051411 (**1S**), and 1051412 (**2**) contain supplementary crystallographic data. The data can be obtained free of charge from The Cambridge Crystallographic Data Centre via http://www.ccdc.cam.ac.uk/data_request/cif.
